# Covariance-Based Estimation for Clustered Sensor Networks Subject to Random Deception Attacks

**DOI:** 10.3390/s19143112

**Published:** 2019-07-14

**Authors:** Raquel Caballero-Águila, Aurora Hermoso-Carazo, Josefa Linares-Pérez

**Affiliations:** 1 Dpto. de Estadística, Universidad de Jaén, Paraje Las Lagunillas, 23071 Jaén, Spain; 2 Dpto. de Estadística, Universidad de Granada, Avda. Fuentenueva, 18071 Granada, Spain

**Keywords:** least-squares filtering, least-squares fixed-point smoothing, networked systems, cluster-based approach, stochastic deception attacks

## Abstract

In this paper, a cluster-based approach is used to address the distributed fusion estimation problem (filtering and fixed-point smoothing) for discrete-time stochastic signals in the presence of random deception attacks. At each sampling time, measured outputs of the signal are provided by a networked system, whose sensors are grouped into clusters. Each cluster is connected to a local processor which gathers the measured outputs of its sensors and, in turn, the local processors of all clusters are connected with a global fusion center. The proposed cluster-based fusion estimation structure involves two stages. First, every single sensor in a cluster transmits its observations to the corresponding local processor, where least-squares local estimators are designed by an innovation approach. During this transmission, deception attacks to the sensor measurements may be randomly launched by an adversary, with known probabilities of success that may be different at each sensor. In the second stage, the local estimators are sent to the fusion center, where they are combined to generate the proposed fusion estimators. The covariance-based design of the distributed fusion filtering and fixed-point smoothing algorithms does not require full knowledge of the signal evolution model, but only the first and second order moments of the processes involved in the observation model. Simulations are provided to illustrate the theoretical results and analyze the effect of the attack success probability on the estimation performance.

## 1. Introduction

Nowadays, communication networks are widely used to cope with signal estimation problems in engineering, economy, health or security, among others, since they generally provide more robust and precise estimators of the target signal than a single sensor. Conventional centralized and distributed fusion estimation architectures have been widely studied under a state-space approach (see e.g., [1,2,3,4] and references therein). In addition, assuming that the evolution model of the signal is not fully known and only covariance information is available, centralized and distributed fusion estimation algorithms have been proposed for sensor networks affected by different network-induced random uncertainties (see e.g., [5,6,7]). A comprehensive survey of recent developments in estimation and fusion for networked systems with randomly occurring phenomena can be found in [8]. The key theories and methodologies of distributed multisensor data fusion are comprehensively reviewed in [9] and a survey of distributed fusion estimation algorithms with applications in networked systems, including an interesting analysis of some network-induced uncertainties, is provided in [10].

These conventional centralized and distributed fusion architectures require connecting all sensor nodes to a central processor, which sometimes can involve a serious communication burden, especially when the number of sensors is large. Additionally, in the distributed fusion estimation architecture, equipping each single sensor with a local estimator may be sometimes unaffordable. To overcome these shortcomings, a usual practice in engineering consists of arranging the sensors in clusters and selecting one node per cluster (called cluster-head node) acting as a local processor. Each cluster head first collects measurements from the regular sensor nodes of its cluster to generate a local estimator and then all local estimators are collected in the central processor, where the distributed fusion estimator is generated. This structure, called hierarchical cluster-based estimation structure, provides an efficient way to analyze big data in a great variety of application fields [11]. Clustering is also widely used, for example, in underwater sensor networks established for military purposes, such as anti-submarine warfare, communications, positioning and guidance [12]. An essential issue when dealing with complex networked systems consisting of multiple clusters or individual subsystems is coordination control and the design of appropriate consensus protocols; relevant results concerning these problems in different kinds of networked systems are found in [13,14,15,16]. A state-of-the-art and comprehensive survey on clustering approaches can be found in [17] and a detailed outline of some modern energy-efficient clustering approaches to improve the lifetime of wireless sensor networks can be seen in [18]. Recently, a comprehensive review with comparisons and classifications of different optimized clustering approaches according to different metrics has been carried out in [19].

A remarkable disadvantage of using sensor networks for signal estimation is that their reliability can be compromised by possible cyber-attacks from adversaries. For this reason, cyber-security of networked systems is drawing considerable research attention. The most typical kinds of attacks are the denial-of-service attacks and the deception attacks [20]. While the first ones strike at data availability by obstructing the flow of information through the network, the second ones violate data integrity by injecting false information that modifies the real data packets. Such false information can include a wrong sensor measurement or control input, an incorrect time-stamp or a wrong identity of the sending device. The fusion estimation problem for stochastic signals from measured data coming from a sensor network subject to deception attacks is currently an important focus of research (see e.g., [20,21,22,23,24,25,26] and references therein). In [20], new insecurity conditions for the state estimation problem under false data injection attacks are proposed. In [21], the variance-constrained distributed filtering problem is studied for time-varying systems subject to multiplicative deception attacks with bounded attack noises. A distributed recursive filtering algorithm is designed in [22] for a class of discrete time-delayed systems subject to both uniform quantization and intermittent deception attacks. In [23], the security-guaranteed filtering problem is addressed for a class of nonlinear discrete time-delayed systems with both stochastic sensor saturations and deception attacks. The centralized security-guaranteed filtering problem for linear discrete time-invariant stochastic systems with multi-rate sensor nodes is addressed in [24], when deception attacks are launched during the transmission of information from the sensors to the centralized filter. In [25], the cluster-based covariance intersection fusion estimation problem under stochastic deception attacks is investigated. An integrated analysis of event-triggered fault detection and fault estimation is proposed in [26] for a class of discrete-time stochastic systems subject to unknown disturbances and stochastic deception attacks.

*Research motivation.* In this paper, distributed fusion filtering and fixed-point smoothing algorithms are designed for clustered sensor networks subject to stochastic linear deception attacks. This study is motivated by the following challenges:

*(i)* To find out fusion estimation structures for multi-sensor networked systems that reduce transmission burdens (with respect to both centralized and distributed fusion) and the cost of embedding a local processor in each sensor (with respect to distributed fusion).

*(ii)* To design recursive and easily implementable estimation algorithms with high estimation accuracy, fusing unreliable multi-sensor data, corrupted by possible deception attacks, under general assumptions on the target signal that do not require the full knowledge of the system state-space model.

*Paper contributions.* In light of our motivations, the main contributions of this paper are highlighted as follows:

*(i)* The fusion estimation problem in multi-sensor networked systems when the sensors are grouped into clusters is investigated assuming, on the one hand, that the measurements are subject to stochastic deception attacks and, on the other, that the signal evolution model is not necessarily known, but only information about its first and second order statistical properties (*covariance information*) is available. This covariance-based estimation approach is more general than the conventional one based on the full knowledge of the state-space model of the system since, in that case, the mean and autocovariance function of the signal process can be calculated. Hence, this estimation approach provides a comprehensive framework to deal with a great variety of stochastic signals.

*(ii)* A two-stage fusion estimation algorithm is designed for both filtering and fixed-point smoothing problems. In the first stage, each local processor collects measurements (subject to random deception attacks) from its cluster to generate local least-squares linear estimators; in this stage, the innovation approach is used to design the local estimation algorithms, which are recursive and computationally simple. In the second stage, in order to improve the estimation performance, all these local estimators are transmitted to the fusion center, where fusion estimators are obtained by matrix-weighted linear combinations of the local estimators; the weight matrices are computed by minimizing the mean squared estimation error, for which the cross-correlation matrices between any two local estimators need to be previously calculated.

In contrast with previous papers concerning the fusion estimation problem under a covariance-based approach, this is the first time that a cluster structure of the sensor network is used and stochastic deception attacks are considered. In comparison with the existing literature about the estimation problem in clustered sensor networks, either subject to random deception attacks or not, the main difference is the kind of information required for the derivation of the algorithms: full knowledge of the state-space model in the existing literature and only covariance information (which covers the conventional formulation based on state-space model and also more general situations) in the current paper.

*Paper structure.* The measurement model framework is presented in Section 2, where the equations of the clustering sensor measurements and the stochastic deception attacks, together with the hypotheses under which the distributed estimation problem will be addressed, are specified. In Section 3 and Section 4 the first and second stages of the proposed cluster-based fusion estimation structure are developed. The local least-squares linear filtering and fixed-point smoothing estimators, as well as formulas for the error covariance matrices, are obtained by recursive algorithms in Section 3. The proposed distributed fusion estimators are proposed in Section 4 by a matrix-weighted linear combination of the local estimators using the mean squared error as optimality criterion; also, to measure the estimation accuracy, recursive formulas for the error covariance matrices are derived. The performance of the proposed estimation algorithms is analyzed in a numerical simulation study, carried out in Section 5. Finally, some concluding remarks are provided in Section 6.

*Notation.* Unless otherwise stated, the notation used in the paper is fairly standard. $R^{n}$ denotes the *n*-dimensional Euclidean space and $R^{m\times n}$ the set of all m×n real matrices. When the dimensions of vectors or matrices are not specified, they are assumed to be compatible with algebraic operations. $1_{n}$ denotes the *n*-dimensional column vector with all ones, while the identity and zero matrices are denoted by *I* and 0, respectively. For any function $\Gamma_{h,k}$, depending on *h* and *k*, and $\Xi^{\left(rs\right)}$, depending on *r* and *s*, we will write $\Gamma_{k}=\Gamma_{k,k}$ and $\Xi^{\left(r\right)}=\Xi^{\left(rr\right)}$, respectively, for simplicity. The Kronecker product of the matrices $A\in R^{m\times n}$ and $B\in R^{m^{\prime }\times n^{\prime }}$, which is a matrix in $R^{mm^{\prime }\times nn^{\prime }}$, is denoted by A⊗B. The Hadamard product of the matrices $C,D\in R^{m\times n}$, which is also a matrix in $R^{m\times n}$, is denoted by C∘D and it is defined by ${\left(C\circ D\right)}_{ij}=C_{ij}D_{ij}$. $\delta_{k,s}$ is the Kronecker delta function, i.e., $\delta_{k,s}=1$ when k=s and zero otherwise. The autocovariance function of a second-order process ${\left\{\alpha_{k}\right\}}_{k\geq 1}$ is defined as $E\left[\left(\alpha_{k}-E\left[\alpha_{k}\right]\right){\left(\alpha_{s}-E\left[\alpha_{s}\right]\right)}^{T}\right],\;k,s\geq 1,$ where E[·] stands for the mathematical expectation operator.

## 2. Estimation Problem Formulation and Measurement Model

In this paper, we consider the distributed fusion estimation problem of a discrete-time stochastic signal from a set of measurements provided by multiple sensors. A cluster-based estimation structure will be adopted: the sensors are grouped into different clusters, each of them connected with a local processor which, in turn, is connected with a global fusion center (FC). The distributed fusion estimation process operates in two stages. In the first one, the measured outputs provided by the sensors of each cluster are sent to the corresponding local processor, where local least-squares (LS) linear estimators are obtained; it is assumed that, during the transmission to the local processors, the clustering sensor measurements are subject to stochastic linear deception attacks. In the second stage, the local estimators received from all the local processors are gathered and fused in the FC, where the proposed distributed signal estimators are generated by a matrix-weighted linear combination of the local estimators using the mean squared error as optimality criterion.

The aforementioned estimation problem will be addressed without requiring full knowledge of the evolution model generating the signal process. Instead, it will be assumed that the signal mean function is zero and its covariance function is factorizable (*covariance-based estimation approach*). More precisely, the following assumption is required ([7]):

***(A1)***  The $n_{x}$-dimensional signal ${\left\{x_{k}\right\}}_{k\geq 1}$ is a zero-mean second-order process and its autocovariance function is expressed in a separable form; namely, $E\left[x_{k}x_{h}^{T}\right]=A_{k}B_{h}^{T},\;h\leq k,$ where $A_{k},B_{h}\in R^{n_{x}\times n}$ are known matrices.

The signal process of the most common signal evolution models (e.g., the signal of linear systems, or that of uncertain systems with a sum of multiple multiplicative noise terms) meets this assumption ***(A1)*** and, hence, the covariance-based estimation approach provides a comprehensive framework to cope with different signal evolution models, thus overcoming the necessity of deriving specific algorithms for each situation.

### 2.1. Clustering Sensor Measurements and Stochastic Deception Attacks

Consider a sensor network and assume that the sensor nodes are grouped into *L* clusters to measure the stochastic signal of interest. Specifically, assume that each cluster r=1,…,L is made up of $m_{r}$ sensors that provide measurements of the signal according to the following model:

(1) $$z_{k}^{\left(r(i)\right)}=C_{k}^{\left(r(i)\right)}x_{k}+v_{k}^{\left(r(i)\right)}\;\;,\;\;\;k\geq 1;\;\;\;\;i=1,\ldots ,m_{r},\;\;\;\;r=1,\ldots ,L,$$

where $C_{k}^{\left(r(i)\right)}$ are known matrices and $z_{k}^{\left(r(i)\right)}\in R^{n_{z}}$ is the signal measured output from the *i*-th sensor of the *r*-th cluster at time *k*, which is transmitted to the *r*-th local processor to obtain the local LS linear signal estimators. The following assumption is required on the measurement noises, ${\left\{v_{k}^{\left(r(i)\right)}\right\}}_{k\geq 1}$, $i=1,\ldots ,m_{r},\;\;r=1,\ldots ,L$:

***(A2)***  *The measurement noises of different clusters are independent and, for each r=1,…,L, the noises ${\left\{v_{k}^{\left(r(i)\right)}\right\}}_{k\geq 1}$, $i=1,\ldots ,m_{r}$, are zero-mean second-order white processes with known covariance matrices*
  $$E\left[v_{k}^{\left(r(i)\right)}v_{h}^{(r(j))T}\right]=R_{k}^{\left(r(ij)\right)}\delta_{k,h},\;\;\;k,h\geq 1;\;\;\;\;i,j=1,\ldots ,m_{r}.$$

For each cluster r=1,…,L, the transmissions of the measured outputs $z_{k}^{\left(r(i)\right)},\;i=1,\ldots ,m_{r},$ to the *r*-th local processor are affected by random linear deception attacks and the deceptive signal injected by the attackers, $\xi_{k}^{\left(r(i)\right)}$, is described by:

(2) $$\xi_{k}^{\left(r(i)\right)}=-z_{k}^{\left(r(i)\right)}+w_{k}^{\left(r(i)\right)},\;\;\;k\geq 1;\;\;\;\;i=1,\ldots ,m_{r},\;\;\;\;r=1,\ldots ,L.$$

This signal involves two parts: the first one neutralizes the true information and the second one is the blurred information (noise) added by the attackers. These noises, ${\left\{w_{k}^{\left(r(i)\right)}\right\}}_{k\geq 1}$, $i=1,\ldots ,m_{r},\;\;r=1,\ldots ,L$, are assumed to satisfy the following requirement:

***(A3)***  *The attack noises of different clusters are independent and, for each r=1,…,L, the noises ${\left\{w_{k}^{\left(r(i)\right)}\right\}}_{k\geq 1}$, $i=1,\ldots ,m_{r}$, are zero-mean second-order white processes with known covariance matrices*
  $$E\left[w_{k}^{\left(r(i)\right)}w_{h}^{(r(j))T}\right]=S_{k}^{\left(r(ij)\right)}\delta_{k,h},\;\;\;k,h\geq 1;\;\;\;\;i,j=1,\ldots ,m_{r}.$$

### 2.2. Measurements Received by the Local Processors

Usually, in practice, the attacks may randomly succeed or not. So, taking into account this random nature of the attacks, for every r=1,…,L, the measurements received by the *r*-th local processor are modelled by introducing different sequences of Bernoulli random variables, ${\left\{\lambda_{k}^{\left(r(i)\right)}\right\}}_{k\geq 1}$, $i=1,\ldots ,m_{r}$. For each r=1,…,L, and $i=1,\ldots ,m_{r}$, the value $\lambda_{k}^{\left(r(i)\right)}=1$ models a successful attack into the *i*-th communication channel from the *r*-th cluster, meaning that only noise $w_{k}^{\left(r(i)\right)}$ arrives to the *r*-th local processor; conversely, the value $\lambda_{k}^{\left(r(i)\right)}=0$ models a failed attack, which means that the real measured output $z_{k}^{\left(r(i)\right)}$ is received by the *r*-th local processor. Taking these considerations into account, the following model for $y_{k}^{\left(r(i)\right)}$, the measurements received by the *r*-th local processor, is considered:

(3) $$y_{k}^{\left(r(i)\right)}=z_{k}^{\left(r(i)\right)}+\lambda_{k}^{\left(r(i)\right)}\xi_{k}^{\left(r(i)\right)}\;\;,\;\;\;k\geq 1;\;\;\;\;i=1,\ldots ,m_{r},\;\;\;\;r=1,\ldots ,L,$$

or, equivalently, by substituting (1) and (2) into (3), we have:

(4) $$y_{k}^{\left(r(i)\right)}=\left(1-\lambda_{k}^{\left(r(i)\right)}\right)\left(C_{k}^{\left(r(i)\right)}x_{k}+v_{k}^{\left(r(i)\right)}\right)+\lambda_{k}^{\left(r(i)\right)}w_{k}^{\left(r(i)\right)}\;\;,\;\;\;k\geq 1;\;\;\;\;i=1,\ldots ,m_{r},\;\;\;\;r=1,\ldots ,L.$$

The following assumption is imposed on the Bernoulli random variables describing the success or failure of attacks:

***(A4)***  ${\left\{\lambda_{k}^{\left(r(i)\right)}\right\}}_{k\geq 1}$, r=1,…,L, $i=1,\ldots ,m_{r}$, are independent sequences of independent Bernoulli random variables with known probabilities $P\left(\lambda_{k}^{\left(r(i)\right)}=1\right)={\bar{\lambda }}_{k}^{\left(r(i)\right)}$.

From this assumption, if we denote $\lambda_{k}^{\left(r\right)}={\left(\lambda_{k}^{\left(r(1)\right)},\ldots ,\lambda_{k}^{\left(r\left(m_{r}\right)\right)}\right)}^{T}⊗1_{n_{z}}$, for r=1,…,L, then the correlation matrices $K_{k}^{\lambda^{\left(r\right)}}\;\;\equiv E\left[\lambda_{k}^{\left(r\right)}\lambda_{k}^{(r)T}\right]$ and $K_{k}^{1-\lambda^{\left(r\right)}}\equiv E\left[\left(1_{m_{r}n_{z}}-\lambda_{k}^{\left(r\right)}\right){\left(1_{m_{r}n_{z}}-\lambda_{k}^{\left(r\right)}\right)}^{T}\right]$ are known and their entries are easily calculated taking into account that

(5) $$E\left[\lambda_{k}^{\left(r(i)\right)}\lambda_{k}^{\left(r(j)\right)}\right]=\left\{\begin{array}{c} {\bar{\lambda }}_{k}^{\left(r(i)\right)},\;\;i=j,\;\\ {\bar{\lambda }}_{k}^{\left(r(i)\right)}{\bar{\lambda }}_{k}^{\left(r(j)\right)},\;\;i\neq j. \end{array}\right.$$

Finally, the following independence hypothesis is also assumed:

***(A5)***  For r=1,…,L and $i=1,\ldots ,m_{r}$, the signal process ${\left\{x_{k}\right\}}_{k\geq 1}$ and the processes ${\left\{v_{k}^{\left(r(i)\right)}\right\}}_{k\geq 1}$, ${\left\{w_{k}^{\left(r(i)\right)}\right\}}_{k\geq 1}$ and ${\left\{\lambda_{k}^{\left(r(i)\right)}\right\}}_{k\geq 1}$ are mutually independent.

## 3. First Stage: Local LS Linear Estimators

To start with, as indicated previously, our aim is to calculate in every local processor, r=1,…,L, LS linear estimators of the signal based on the measurements received from all the sensors of the *r*-th cluster. Therefore, to estimate the signal $x_{k}$ in the local processor *r* at time k+N, we consider all the measurements received from all the sensors $i=1,\ldots ,m_{r}$, of the *r*-th cluster, up to time k+N; that is, the measurement set $\left\{y_{h}^{\left(r(i)\right)}\;,\;\;h\leq k+N,\;\;i=1,\ldots ,m_{r}\right\}$. So defining the vectors $y_{h}^{\left(r\right)}={\left(y_{h}^{(r(1))T}\;\;,\ldots ,y_{h}^{(r\left(m_{r}\right))T}\right)}^{T}$, made up of all the measurements received by the *r*-th processor at each sampling time *h*, the problem at hand is formulated as that of determining local LS linear estimators of the signal $x_{k}$ based on the vectors $\left\{y_{h}^{\left(r\right)}\;,\;\;h\leq k+N\right\}$, for r=1,…,L.

### 3.1. Stacked Model for the Measurements Received by the Local Processors

Taking into account Equation (4) for the measurements received by the *r*-th local processor, the following model for the above-defined vectors $y_{k}^{\left(r\right)}$ is clearly deduced:

(6) $$y_{k}^{\left(r\right)}=\left(I-\Lambda_{k}^{\left(r\right)}\right)\left(C_{k}^{\left(r\right)}x_{k}+v_{k}^{\left(r\right)}\right)+\Lambda_{k}^{\left(r\right)}w_{k}^{\left(r\right)}\;\;,\;\;\;k\geq 1;\;\;\;\;r=1,\ldots ,L,$$

where

$$C_{k}^{\left(r\right)}\;=\;\left(\;\begin{array}{c} C_{k}^{\left(r(1)\right)}\\ \vdots \\ C_{k}^{\left(r\left(m_{r}\right)\right)} \end{array}\;\right),\;\;v_{k}^{\left(r\right)}\;=\;\left(\;\begin{array}{c} v_{k}^{\left(r(1)\right)}\\ \vdots \\ v_{k}^{\left(r\left(m_{r}\right)\right)} \end{array}\;\right),\;\;w_{k}^{\left(r\right)}\;=\;\left(\;\begin{array}{c} w_{k}^{\left(r(1)\right)}\\ \vdots \\ w_{k}^{\left(r\left(m_{r}\right)\right)} \end{array}\;\right),\;\;\Lambda_{k}^{\left(r\right)}\;=\;\left(\;\begin{array}{ccc} \lambda_{k}^{\left(r(1)\right)} & \cdots & 0\\ \vdots & \ddots & \vdots \\ 0 & \cdots & \lambda_{k}^{\left(r\left(m_{r}\right)\right)} \end{array}\;\right)⊗I.$$

The following statistical properties of the processes involved in model (6), which will be used to address the LS linear estimation problem, are easily inferred from the model assumptions ***(A1)–(A5)*** stated in Section 2:

***(P1)***  ${\left\{v_{k}^{\left(r\right)}\right\}}_{k\geq 1}$, r=1,…,L, are independent zero-mean noise processes with
  $$E\left[v_{k}^{\left(r\right)}v_{h}^{\left(s\right)}\right]=R_{k}^{\left(r\right)}\delta_{k,h}\delta_{r,s},\;\;\;\mathrm{where}\;\;\;R_{k}^{\left(r\right)}={\left(R_{k}^{\left(r(ij)\right)}\right)}_{i,j=1,\ldots ,m_{r}}.$$
***(P2)***  ${\left\{w_{k}^{\left(r\right)}\right\}}_{k\geq 1}$, r=1,…,L, are independent zero-mean noise processes with
  $$E\left[w_{k}^{\left(r\right)}w_{h}^{\left(s\right)}\right]=S_{k}^{\left(r\right)}\delta_{k,h}\delta_{r,s},\;\;\;\mathrm{where}\;\;\;S_{k}^{\left(r\right)}={\left(S_{k}^{\left(r(ij)\right)}\right)}_{i,j=1,\ldots ,m_{r}}.$$
***(P3)***  ${\left\{\Lambda_{k}^{\left(r\right)}\right\}}_{k\geq 1}$, r=1,…,L, are independent sequences of independent random matrices with known means, ${\bar{\Lambda }}_{k}^{\left(r\right)}=Diag\left({\bar{\lambda }}_{k}^{\left(r(1)\right)},\ldots ,{\bar{\lambda }}_{k}^{\left(r\left(m_{r}\right)\right)}\right)⊗I.$ Moreover, the Hadamard product properties guarantee that, for any random matrix *G* independent of $\Lambda_{k}^{\left(r\right)}$, $E\left[\Lambda_{k}^{\left(r\right)}G\Lambda_{k}^{\left(r\right)}\right]=K_{k}^{\lambda^{\left(r\right)}}\circ E\left[G\right]$, where $K_{k}^{\lambda^{\left(r\right)}}$ is calculated from (5).
***(P4)***  For r=1,…,L, the signal process, ${\left\{x_{k}\right\}}_{k\geq 1}$, and the processes ${\left\{v_{k}^{\left(r\right)}\right\}}_{k\geq 1}$, ${\left\{w_{k}^{\left(r\right)}\right\}}_{k\geq 1}$ and ${\left\{\Lambda_{k}^{\left(r\right)}\right\}}_{k\geq 1}$ are mutually independent.
***(P5)***  ${\left\{y_{k}^{\left(r\right)}\right\}}_{k\geq 1}$, r=1,…,L, are zero-mean processes with covariance matrices $\Sigma_{k}^{y^{\left(rs\right)}}\equiv E\left[y_{k}^{\left(r\right)}y_{k}^{(s)T}\right]$ given by
  (7) $$\Sigma_{k}^{y^{\left(rs\right)}}=\left\{\begin{array}{cc} K_{k}^{1-\lambda^{\left(r\right)}}\circ \left(C_{k}^{\left(r\right)}A_{k}B_{k}^{T}C_{k}^{(r)T}+R_{k}^{\left(r\right)}\right)+K_{k}^{\lambda^{\left(r\right)}}\circ S_{k}^{\left(r\right)}, & r=s,\\ \left(I-{\bar{\Lambda }}_{k}^{\left(r\right)}\right)C_{k}^{\left(r\right)}A_{k}B_{k}^{T}C_{k}^{(s)T}\left(I-{\bar{\Lambda }}_{k}^{\left(s\right)}\right), & r\neq s, \end{array}\right.$$
  where $K_{k}^{1-\lambda^{\left(r\right)}}$ and $K_{k}^{\lambda^{\left(r\right)}}$ are calculated from (5).

### 3.2. Recursive Local LS Linear Filtering and Fixed-Point Smoothing Algorithms

This subsection is devoted to the design of recursive algorithms, at each local processor r=1,…,L, for the LS linear filtering and fixed-point smoothing estimators based on the measurements received from all the sensors of the *r*-th cluster. In other words, for the *r*-th cluster, r=1,…,L, the aim in this subsection is the design of algorithms to obtain the local LS linear estimators, ${\hat{x}}_{k/k+N}^{\left(r\right)}$, N≥0, of the signal $x_{k}$ based on the vectors $y_{h}^{\left(r\right)}\;\;,\;h\leq k+N,$ given by (6); specifically, a recursive algorithm for the local LS filter ${\hat{x}}_{k/k}^{\left(r\right)}$, k≥1, and a recursive algorithm for the local LS smoother ${\hat{x}}_{k/k+N}^{\left(r\right)}$, for fixed k≥1 and N=1,2,…, will be derived.

For this purpose, we will use the measurement innovations rather than the raw measurements (innovation approach), where the innovation at time *k* is defined as $\mu_{k}^{\left(r\right)}\equiv y_{k}^{\left(r\right)}-{\hat{y}}_{k/k-1}^{\left(r\right)}$, being ${\hat{y}}_{k/k-1}^{\left(r\right)}$ the one-stage observation predictor (LS linear estimator of $y_{k}^{\left(r\right)}$ based on $y_{h}^{\left(r\right)}\;\;,\;\;h\leq k-1$). Taking into account the properties of the innovation process, the following expression for the LS linear estimators is derived:

(8) $${\hat{x}}_{k/H}^{\left(r\right)}=\sum_{h=1}^{H}E\left[x_{k}\mu_{h}^{(r)T}\right]{\left(E\left[\mu_{h}^{\left(r\right)}\mu_{h}^{(r)T}\right]\right)}^{-1}\mu_{h}^{\left(r\right)}.$$

Taking orthogonal projections in (6) and using properties ***(P2)–(P4)***, we get that the one-stage observation predictor is ${\hat{y}}_{h/h-1}^{\left(r\right)}=\left(I-{\bar{\Lambda }}_{h}^{\left(r\right)}\right)C_{h}^{\left(r\right)}{\hat{x}}_{h/h-1}^{\left(r\right)}$ and, consequently, the innovation is given by

(9) $$\mu_{h}^{\left(r\right)}=y_{h}^{\left(r\right)}-\left(I-{\bar{\Lambda }}_{h}^{\left(r\right)}\right)C_{h}^{\left(r\right)}{\hat{x}}_{h/h-1}^{\left(r\right)}.$$

Furthermore, from property ***(P5)***, it is clear that the innovation covariance matrix, $\Pi_{h}^{\left(r\right)}\equiv E\left[\mu_{h}^{\left(r\right)}\mu_{h}^{(r)T}\right]=E\left[y_{h}^{\left(r\right)}y_{h}^{(r)T}\right]-E\left[{\hat{y}}_{h/h-1}^{\left(r\right)}{\hat{y}}_{h/h-1}^{(r)T}\right]$, satisfies

$$\Pi_{h}^{\left(r\right)}=\Sigma_{h}^{y^{\left(r\right)}}-\left(I-{\bar{\Lambda }}_{h}^{\left(r\right)}\right)C_{h}^{\left(r\right)}E\left[{\hat{x}}_{h/h-1}^{\left(r\right)}{\hat{x}}_{h/h-1}^{(r)T}\right]C_{h}^{(r)T}\left(I-{\bar{\Lambda }}_{h}^{\left(r\right)}\right).$$

Using now (6) for $y_{h}^{\left(r\right)}$ and (8) for ${\hat{x}}_{h/h-1}^{\left(r\right)}$, we obtain that the coefficients $X_{k,h}^{\left(r\right)}=E\left[x_{k}\mu_{h}^{(r)T}\right]$ satisfy $X_{k,h}^{\left(r\right)}=A_{k}O_{h}^{\left(r\right)},\;\;h\leq k$, where $O_{h}^{\left(r\right)}$ is a matrix function such that

(10) $$O_{h}^{\left(r\right)}=\left(B_{h}^{T}-\sum_{l=1}^{h-1}O_{l}^{\left(r\right)}\Pi_{l}^{(r)-1}O_{l}^{(r)T}A_{h}^{T}\right)C_{h}^{(r)T}\left(I-{\bar{\Lambda }}_{h}^{\left(r\right)}\right).$$

Then, by defining

(11) $$\begin{array}{c} o_{k}^{\left(r\right)}=\left(1-\delta_{k,0}\right)\sum_{h=1}^{k}O_{h}^{\left(r\right)}\Pi_{h}^{(r)-1}\mu_{h}^{\left(r\right)},\;\;\;k\geq 1,\\ \Sigma_{k}^{o^{\left(r\right)}}=\left(1-\delta_{k,0}\right)\sum_{h=1}^{k}O_{h}^{\left(r\right)}\Pi_{h}^{(r)-1}O_{h}^{(r)T}\;\;,\;\;\;k\geq 1, \end{array}$$

we obtain that the estimators ${\hat{x}}_{k/L}^{\left(r\right)},\;L\leq k$, are given by ${\hat{x}}_{k/L}^{\left(r\right)}=A_{k}o_{L}^{\left(r\right)}$, and, by substitution in (9), we obtain that $\mu_{h}^{\left(r\right)}=y_{h}^{\left(r\right)}-\left(I-{\bar{\Lambda }}_{h}^{\left(r\right)}\right)C_{h}^{\left(r\right)}A_{h}o_{h-1}^{\left(r\right)}$.

Finally, since (11) guarantees that $\sum_{l=1}^{h-1}O_{l}^{\left(r\right)}\Pi_{l}^{(r)-1}O_{l}^{(r)T}=\Sigma_{h-1}^{o^{\left(r\right)}}$, by substitution in (10), it is deduced that $O_{h}^{\left(r\right)}=\left(B_{h}^{T}-\Sigma_{h-1}^{o^{\left(r\right)}}A_{h}^{T}\right)C_{h}^{(r)T}\left(I-{\bar{\Lambda }}_{h}^{\left(r\right)}\right)$. Bearing in mind the above results, the following filtering algorithm is derived.

***Recursive Local LS Linear Filtering Algorithm.*** *For the r-th cluster, r=1,…,L, the local filtering estimators, ${\hat{x}}_{k/k}^{\left(r\right)}$, and the error covariance matrices, ${\hat{\Sigma }}_{k/k}^{\left(r\right)}\equiv E\left[\left(x_{k}-{\hat{x}}_{k/k}^{\left(r\right)}\right){\left(x_{k}-{\hat{x}}_{k/k}^{\left(r\right)}\right)}^{T}\right]$, are recursively obtained by*

(12) $$\begin{array}{c} {\hat{x}}_{k/k}^{\left(r\right)}=A_{k}o_{k}^{\left(r\right)},\;\;\;k\geq 1,\\ {\hat{\Sigma }}_{k/k}^{\left(r\right)}=A_{k}{\left(B_{k}-A_{k}\Sigma_{k}^{o^{\left(r\right)}}\right)}^{T}\;\;,\;\;\;k\geq 1. \end{array}$$

*The vectors $o_{k}^{\left(r\right)}$ and the matrices $\Sigma_{k}^{o^{\left(r\right)}}\equiv E\left[o_{k}^{\left(r\right)}o_{k}^{(r)T}\right]$, defined in (11), are recursively calculated from*

(13) $$o_{k}^{\left(r\right)}=o_{k-1}^{\left(r\right)}+O_{k}^{\left(r\right)}\Pi_{k}^{(r)-1}\mu_{k}^{\left(r\right)},\;\;\;k\geq 1;\;\;\;\;o_{0}^{\left(r\right)}=0,$$

$$\Sigma_{k}^{o^{\left(r\right)}}=\Sigma_{k-1}^{o^{\left(r\right)}}+O_{k}^{\left(r\right)}\Pi_{k}^{(r)-1}O_{k}^{(r)T}\;\;,\;\;\;k\geq 1;\;\;\;\;\Sigma_{0}^{o^{\left(r\right)}}=0.$$

*The matrices $O_{k}^{\left(r\right)}\equiv E\left[o_{k}^{\left(r\right)}\mu_{k}^{(r)T}\right]$, given in (10), satisfy*

$$O_{k}^{\left(r\right)}={\left(B_{k}-A_{k}\Sigma_{k-1}^{o^{\left(r\right)}}\right)}^{T}C_{k}^{(r)T}\left(I-{\bar{\Lambda }}_{k}^{\left(r\right)}\right),\;\;\;k\geq 1.$$

*The innovations, $\mu_{k}^{\left(r\right)}=y_{k}^{\left(r\right)}-{\hat{y}}_{k/k-1}^{\left(r\right)}$, are given by*

(14) $$\mu_{k}^{\left(r\right)}=y_{k}^{\left(r\right)}-\left(I-{\bar{\Lambda }}_{k}^{\left(r\right)}\right)C_{k}^{\left(r\right)}A_{k}o_{k-1}^{\left(r\right)},\;\;\;k\geq 1,$$

*and the innovation covariance matrices, $\Pi_{k}^{\left(r\right)}\equiv E\left[\mu_{k}^{\left(r\right)}\mu_{k}^{(r)T}\right]$, satisfy*

$$\Pi_{k}^{\left(r\right)}=\Sigma_{k}^{y^{\left(r\right)}}-\left(I-{\bar{\Lambda }}_{k}^{\left(r\right)}\right)C_{k}^{\left(r\right)}A_{k}\Sigma_{k-1}^{o^{\left(r\right)}}A_{k}^{T}C_{k}^{(r)T}\left(I-{\bar{\Lambda }}_{k}^{\left(r\right)}\right),\;\;\;k\geq 1,$$

*where the matrices $\Sigma_{k}^{y^{\left(r\right)}}$ are given in (7).*

The general expression (8) for the LS linear estimators is also the starting point to derive the following covariance-based recursive fixed-point smoothing algorithm. The derivation is omitted, since this algorithm can easily be deduced by an analogous reasoning to that used in Theorem 2 of [6].

***Recursive Local LS Linear Fixed-point Smoothing Algorithm.*** *For the r-th cluster, r=1,…,L, starting from the filter, ${\hat{x}}_{k/k}^{\left(r\right)}$, the local LS linear fixed-point smoothers, ${\hat{x}}_{k/k+N}^{\left(r\right)}$, N≥1, are calculated as*

(15) $${\hat{x}}_{k/k+N}^{\left(r\right)}={\hat{x}}_{k/k+N-1}^{\left(r\right)}+X_{k,k+N}^{\left(r\right)}\Pi_{k+N}^{(r)-1}\mu_{k+N}^{\left(r\right)},\;\;\;N\geq 1,\;\;\;k\geq 1,$$

*where the matrices $X_{k,k+N}^{\left(r\right)}\equiv E\left[x_{k}\mu_{k+N}^{(r)T}\right]$ are recursively calculated by*

$$X_{k,k+N}^{\left(r\right)}=\left(B_{k}-M_{k,k+N-1}^{\left(r\right)}\right)A_{k+N}^{T}C_{k+N}^{(r)T}\left(I-{\bar{\Lambda }}_{k+N}^{\left(r\right)}\right),\;\;\;N\geq 1,\;\;\;k\geq 1,$$

*with initial condition $X_{k,k}^{\left(r\right)}=A_{k}O_{k}^{\left(r\right)},\;k\geq 1.$ The matrices $M_{k,k+N}^{\left(r\right)}\equiv E\left[x_{k}o_{k+N}^{(r)T}\right]$, of the expression of $X_{k,k+N}^{\left(r\right)}$, obey the following recursive formula*

$$\begin{array}{c} M_{k,k+N}^{\left(r\right)}=M_{k,k+N-1}^{\left(r\right)}+X_{k,k+N}^{\left(r\right)}\Pi_{k+N}^{(r)-1}O_{k+N}^{(r)T},\;\;\;N\geq 1,\;\;\;k\geq 1;\\ M_{k,k}^{\left(r\right)}=A_{k}K_{k}^{o^{\left(r\right)}}\;\;,\;\;\;k\geq 1. \end{array}$$

*Starting from the error covariance matrix of the filter ${\hat{\Sigma }}_{k/k}^{\left(r\right)}$, the fixed-point smoothing error covariance matrices,*

$${\hat{\Sigma }}_{k/k+N}^{\left(r\right)}\equiv E\left[\left(x_{k}-{\hat{x}}_{k/k+N}^{\left(r\right)}\right){\left(x_{k}-{\hat{x}}_{k/k+N}^{\left(r\right)}\right)}^{T}\right]=E\left[x_{k}x_{k}^{T}\right]-E\left[{\hat{x}}_{k/k+N}^{\left(r\right)}{\hat{x}}_{k/k+N}^{(r)T}\right],$$

*are recursively obtained by*

$${\hat{\Sigma }}_{k/k+N}^{\left(r\right)}={\hat{\Sigma }}_{k/k+N-1}^{\left(r\right)}-X_{k,k+N}^{\left(r\right)}\Pi_{k+N}^{(r)-1}X_{k,k+N}^{(r)T},\;\;\;\;N\geq 1,\;\;\;\;k\geq 1.$$

## 4. Second Stage: Distributed Signal Estimators

As we have already mentioned, once the local LS linear estimators, ${\hat{x}}_{k/k+N}^{\left(r\right)}$, r=1,…,L, have been obtained, they are sent to the FC and our goal is to fuse these local estimators to obtain distributed estimators, ${\hat{x}}_{k/k+N}$, N≥0, as matrix-weighted linear combinations that minimize the mean squared estimation error.

Defining the stacked vectors by comprising all the local estimators, ${\hat{X}}_{k/k+N}={\left({\hat{x}}_{k/k+N}^{(1)T},\ldots ,{\hat{x}}_{k/k+N}^{(L)T}\right)}^{T}$, and applying the LS criterion, the proposed distributed fusion estimators are given by:

(16) $${\hat{x}}_{k/k+N}=E\left[x_{k}{\hat{X}}_{k/k+N}^{T}\right]{\left(E\left[{\hat{X}}_{k/k+N}{\hat{X}}_{k/k+N}^{T}\right]\right)}^{-1}{\hat{X}}_{k/k+N},\;\;\;\;N\geq 0,\;\;\;\;k\geq 1,$$

where $E\left[{\hat{X}}_{k/k+N}{\hat{X}}_{k/k+N}^{T}\right]={\left(E\left[{\hat{x}}_{k/k+N}^{\left(r\right)}{\hat{x}}_{k/k+N}^{(s)T}\right]\right)}_{r,s=1,\ldots ,L}$ and, from the Orthogonal Projection Lemma (OPL), $E\left[x_{k}{\hat{X}}_{k/k+N}^{T}\right]=\left(E\left[{\hat{x}}_{k/k+N}^{\left(1\right)}{\hat{x}}_{k/k+N}^{(1)T}\right],\ldots ,E\left[{\hat{x}}_{k/k+N}^{\left(L\right)}{\hat{x}}_{k/k+N}^{(L)T}\right]\right)$. Hence, the derivation of the distributed estimators in (16) requires to obtain the cross-covariance matrices between the local ones $\Sigma_{k/k+N}^{{\hat{x}}^{\left(rs\right)}}\equiv E\left[{\hat{x}}_{k/k+N}^{\left(r\right)}{\hat{x}}_{k/k+N}^{(s)T}\right],\;r,s=1,\ldots ,L,\;\;k\geq 1,\;\;N\geq 0$. Since the initial condition of the recursive Formula (15) for the local smoothing estimators is the local filter, the cross-covariance matrices, $\Sigma_{k/k+N}^{{\hat{x}}^{\left(rs\right)}},\;N\geq 1,$ between the local smoothers will be recursively obtained by starting from the cross-covariance matrices, $\Sigma_{k/k}^{{\hat{x}}^{\left(rs\right)}}$, between the local filters.

### 4.1. Cross-Covariance Matrices between Local Filtering Estimators $\Sigma_{k/k}^{{\hat{x}}^{\left(rs\right)}}=E\left[{\hat{x}}_{k/k}^{\left(r\right)}{\hat{x}}_{k/k}^{(s)T}\right]$

From expression (12) for the filter ${\hat{x}}_{k/k}^{\left(r\right)}$, denoting $\Sigma_{k}^{o^{\left(rs\right)}}\equiv E\left[o_{k}^{\left(r\right)}o_{k}^{(s)T}\right],$ it is clear that the cross-covariance matrices between any two local filtering estimators ${\hat{x}}_{k/k}^{\left(r\right)}$ and ${\hat{x}}_{k/k}^{\left(s\right)}$ satisfy:

(17) $$\Sigma_{k/k}^{{\hat{x}}^{\left(rs\right)}}=A_{k}\Sigma_{k}^{o^{\left(rs\right)}}A_{k}^{T},\;\;\;\;k\geq 1;\;\;\;\;r,s=1,\ldots ,L.$$

- Using (13) for $o_{k}^{\left(r\right)}$ and denoting $O_{h,k}^{\left(rs\right)}\equiv E\left[o_{h}^{\left(r\right)}\mu_{k}^{(s)T}\right]$, for h=k−1,k, we get that $\Sigma_{k}^{o^{\left(rs\right)}}$ is recursively obtained by:
  $$\Sigma_{k}^{o^{\left(rs\right)}}=\Sigma_{k-1}^{o^{\left(rs\right)}}+O_{k-1,k}^{\left(rs\right)}\Pi_{k}^{(s)-1}O_{k}^{(s)T}+O_{k}^{\left(r\right)}\Pi_{k}^{(r)-1}O_{k}^{(sr)T},\;\;\;k\geq 1,\;\;\;\Sigma_{0}^{o^{\left(rs\right)}}=0;\;\;\;\;r,s=1,\ldots ,L.$$
- Using again (13) and denoting $\Pi_{k}^{\left(rs\right)}\equiv E\left[\mu_{k}^{\left(r\right)}\mu_{k}^{(s)T}\right]$, the following expression for $O_{k}^{\left(rs\right)}=E\left[o_{k}^{\left(r\right)}\mu_{k}^{(s)T}\right]$ is immediately obtained:
  $$O_{k}^{\left(rs\right)}=O_{k-1,k}^{\left(rs\right)}+O_{k}^{\left(r\right)}\Pi_{k}^{(r)-1}\Pi_{k}^{\left(rs\right)},\;\;\;k\geq 1;\;\;\;\;r,s=1,\ldots ,L.$$
- Next, we derive an expression for $O_{k-1,k}^{\left(rs\right)}=E\left[o_{k-1}^{\left(r\right)}\mu_{k}^{(s)T}\right]$. Using (14) for $\mu_{k}^{\left(s\right)}$, with (6) for $y_{k}^{\left(s\right)}$, and taking into account that, from the OPL, $E\left[o_{k-1}^{\left(r\right)}x_{k}\right]=E\left[o_{k-1}^{\left(r\right)}{\hat{x}}_{k/k-1}^{(r)T}\right]$, we obtain:
  $$O_{k-1,k}^{\left(rs\right)}=\left(\Sigma_{k-1}^{o^{\left(r\right)}}-\Sigma_{k-1}^{o^{\left(rs\right)}}\right)A_{k}^{T}C_{k}^{(s)T}\left(I-{\bar{\Lambda }}_{k}^{\left(s\right)}\right),\;\;\;k\geq 1;\;\;\;r,s=1,\ldots ,L$$
- Finally, an expression for the innovation cross-covariance matrices, $\Pi_{k}^{\left(rs\right)}$, is derived. First we write
  $$\Pi_{k}^{\left(rs\right)}=E\left[y_{k}^{\left(r\right)}y_{k}^{(s)T}\right]-E\left[y_{k}^{\left(r\right)}{\hat{y}}_{k/k-1}^{(s)T}\right]-E\left[{\hat{y}}_{k/k-1}^{\left(r\right)}y_{k}^{(s)T}\right]+E\left[{\hat{y}}_{k/k-1}^{\left(r\right)}{\hat{y}}_{k/k-1}^{(s)T}\right].$$
  Next, for t=r,s, we use (6) for $y_{k}^{\left(t\right)}$ and (14) for ${\hat{y}}_{k/k-1}^{\left(t\right)}$; then, using again that, from the OPL, $E\left[x_{k}o_{k-1}^{(t)T}\right]=E\left[{\hat{x}}_{k/k-1}^{\left(t\right)}o_{k-1}^{(t)T}\right]$, the following expression is clear:
  $$\Pi_{k}^{\left(rs\right)}=\Sigma_{k}^{y^{\left(rs\right)}}+\left(I-{\bar{\Lambda }}_{k}^{\left(r\right)}\right)C_{k}^{\left(r\right)}A_{k}\left(\Sigma_{k-1}^{o^{\left(rs\right)}}-\Sigma_{k-1}^{o^{\left(r\right)}}-\Sigma_{k-1}^{o^{\left(s\right)}}\right)A_{k}^{T}C_{k}^{(s)T}\left(I-{\bar{\Lambda }}_{k}^{\left(s\right)}\right),\;\;k\geq 1;\;\;\;r,s=1,\ldots ,L,$$
  where the matrices $\Sigma_{k}^{y^{\left(rs\right)}}$ are given in (7).

### 4.2. Cross-Covariance Matrices between Local Smoothing Estimators $\Sigma_{k/k+N}^{{\hat{x}}^{\left(rs\right)}}=E\left[{\hat{x}}_{k/k+N}^{\left(r\right)}{\hat{x}}_{k/k+N}^{(s)T}\right]$

From expression (15), denoting $\Phi_{k,k+N}^{\left(rs\right)}\equiv E\left[{\hat{x}}_{k/k+N-1}^{\left(r\right)}\mu_{k+N}^{(s)T}\right]$, it is clear that the cross-covariance matrices between any two local smoothing estimators ${\hat{x}}_{k/k+N}^{\left(r\right)}$ and ${\hat{x}}_{k/k+N}^{\left(s\right)}$ satisfy:

(18) $$\begin{array}{c} \Sigma_{k/k+N}^{{\hat{x}}^{\left(rs\right)}}=\Sigma_{k/k+N-1}^{{\hat{x}}^{\left(rs\right)}}+\Phi_{k,k+N}^{\left(rs\right)}\Pi_{k+N}^{(s)-1}X_{k,k+N}^{(s)T}\\ \;+X_{k,k+N}^{\left(r\right)}\Pi_{k+N}^{(r)-1}{\left(\Phi_{k,k+N}^{\left(sr\right)}+X_{k,k+N}^{\left(s\right)}\Pi_{k+N}^{(s)-1}\Pi_{k+N}^{(rs)T}\right)}^{T}\;\;\;,\;\;N\geq 1,\;\;k\geq 1;\;\;\;r,s=1,\ldots ,L. \end{array}$$

- To obtain the expectations $\Phi_{k,k+N}^{\left(rs\right)}\equiv E\left[{\hat{x}}_{k/k+N-1}^{\left(r\right)}\mu_{k+N}^{(s)T}\right]$, we use (14) for $\mu_{k+N}^{\left(s\right)}$ and denote $\Psi_{k,k+N}^{\left(rs\right)}\equiv E\left[{\hat{x}}_{k/k+N}^{\left(r\right)}o_{k+N}^{(s)T}\right]$; then, we have:
  $$\Phi_{k,k+N}^{\left(rs\right)}=E\left[{\hat{x}}_{k/k+N-1}^{\left(r\right)}y_{k+N}^{(s)T}\right]-\Psi_{k,k+N-1}^{\left(rs\right)}A_{k+N}^{T}C_{k+N}^{(s)T}\left(I-{\bar{\Lambda }}_{k+N}^{\left(s\right)}\right).$$
  Using now (6) for $y_{k+N}^{\left(s\right)}$ and the independence of ${\hat{x}}_{k/k+N-1}^{\left(r\right)}$ with $\Lambda_{k+N}^{\left(s\right)}$, $v_{k+N}^{\left(s\right)}$ and $w_{k+N}^{\left(s\right)}$, we have
  $$E\left[{\hat{x}}_{k/k+N-1}^{\left(r\right)}y_{k+N}^{(s)T}\right]=E\left[{\hat{x}}_{k/k+N-1}^{\left(r\right)}x_{k+N}^{T}\right]C_{k+N}^{(s)T}\left(I-{\bar{\Lambda }}_{k+N}^{\left(s\right)}\right),\;\;N\geq 1,\;\;k\geq 1;\;\;\;\;r,s=1,\ldots ,L.$$
  Next, we use that, from the OPL, the estimator is orthogonal to the estimation error, to write $E\left[{\hat{x}}_{k/k+N-1}^{\left(r\right)}x_{k+N}^{T}\right]=E\left[{\hat{x}}_{k/k+N-1}^{\left(r\right)}{\hat{x}}_{k+N/k+N-1}^{(r)T}\right]$. So, since ${\hat{x}}_{k+N/k+N-1}^{\left(r\right)}=A_{k+N}o_{k+N-1}^{\left(r\right)}$, the following expression for $\Phi_{k,k+N}^{\left(rs\right)}$ is immediately derived:
  $$\Phi_{k,k+N}^{\left(rs\right)}=\left(\Psi_{k,k+N-1}^{\left(r\right)}-\Psi_{k,k+N-1}^{\left(rs\right)}\right)A_{k+N}^{T}C_{k+N}^{(s)T}\left(I-{\bar{\Lambda }}_{k+N}^{\left(s\right)}\right),\;\;N\geq 1,\;\;k\geq 1;\;\;\;\;r,s=1,\ldots ,L.$$
- Finally, the expectations $\Psi_{k,k+N}^{\left(rs\right)}=E\left[{\hat{x}}_{k/k+N}^{\left(r\right)}o_{k+N}^{(s)T}\right]$ are calculated. Using (15) for ${\hat{x}}_{k/k+N}^{\left(r\right)}$ and (13) for $o_{k+N}^{\left(s\right)}$, the following expression for $\Psi_{k,k+N}^{\left(rs\right)}$ is clear:
  $$\begin{array}{c} \Psi_{k,k+N}^{\left(rs\right)}=\Psi_{k,k+N-1}^{\left(rs\right)}+\Phi_{k,k+N}^{\left(rs\right)}\Pi_{k+N}^{(s)-1}O_{k+N}^{(s)T}\\ \;+X_{k,k+N}^{\left(r\right)}\Pi_{k+N}^{(r)-1}{\left(O_{k+N-1,k+N}^{\left(sr\right)}+O_{k+N}^{\left(s\right)}\Pi_{k+N}^{(s)-1}\Pi_{k+N}^{(rs)T}\right)}^{T}\;\;\;,\;\;N\geq 1,\;\;k\geq 1;\;\;\;\;r,s=1,\ldots ,L.\\ \Psi_{k,k}^{\left(rs\right)}=A_{k}\Sigma_{k}^{o^{\left(rs\right)}},\;\;k\geq 1;\;\;\;\;r,s=1,\ldots ,L. \end{array}$$

### 4.3. Distributed Filtering and Fixed-Point Smoothing Estimators

The distributed estimators, ${\hat{x}}_{k/k+N}$, are calculated from (16), while a formula for the error covariance matrices, ${\hat{\Sigma }}_{k/k+N}\equiv E\left[\left(x_{k}-{\hat{x}}_{k/k+N}\right){\left(x_{k}-{\hat{x}}_{k/k+N}\right)}^{T}\right]$, is easily derived from assumption ***(A1)*** and (16). More specifically, we can state the following results:

*Let ${\hat{X}}_{k/k+N}={\left({\hat{x}}_{k/k+N}^{(1)T},\ldots ,{\hat{x}}_{k/k+N}^{(L)T}\right)}^{T}$ be the vectors constituted by the local estimators calculated from the recursive algorithms in Section 3.2; then, the distributed filtering and smoothing estimators are given by*

$${\hat{x}}_{k/k+N}=\Xi_{k/k+N}{\left(\Sigma_{k/k+N}\right)}^{-1}{\hat{X}}_{k/k+N},\;\;\;N\geq 0,\;\;\;k\geq 1,$$

*with $\Sigma_{k/k+N}={\left(\Sigma_{k/k+N}^{{\hat{x}}^{\left(rs\right)}}\right)}_{r,s=1,\ldots ,L}$ and $\Xi_{k/k+N}=\left(\Sigma_{k/k+N}^{{\hat{x}}^{\left(1\right)}},\ldots ,\Sigma_{k/k+N}^{{\hat{x}}^{\left(L\right)}}\right),$ where $\Sigma_{k/k+N}^{{\hat{x}}^{\left(rs\right)}}=E\left[{\hat{x}}_{k/k+N}^{\left(r\right)}{\hat{x}}_{k/k+N}^{(s)T}\right],\;r,s=1,\ldots ,L$, are obtained by (18)) for N≥1, with initial condition given in (17).*

*The error covariance matrices of the distributed estimators are computed by*

$${\hat{\Sigma }}_{k/k+N}=A_{k}B_{k}^{T}-\Xi_{k/k+N}\Sigma_{k/k+N}^{-1}\Xi_{k/k+N}^{T},\;\;\;N\geq 0,\;\;\;k\geq 1.$$

## 5. Numerical Simulation Study

A numerical simulation example is presented to illustrate the application of the algorithms designed in the current paper to estimate a two-dimensional signal. Specifically, such algorithms have been implemented by using MATLAB software and both local and distributed filtering and fixed-point smoothing error variances of the signal components have been calculated, for 100 iterations, in order to examine the estimation accuracy. The effect of the stochastic deception attacks on the estimation performance has also been analyzed.

Consider a two-dimensional signal, $x_{k}$, whose evolution is described by the following model:

$$x_{k+1}=\left(F_{1}+\varepsilon_{k}F_{2}\right)x_{k}+G\alpha_{k},\;\;\;\;k\geq 0,$$

where

$$F_{1}=\;\left(\begin{array}{cc} \;0.95 & 0.01\\ \;0 & 0.95 \end{array}\;\right),\;\;\;F_{2}=\;\left(\begin{array}{cc} \;0.01 & 0\\ \;0 & 0.01 \end{array}\;\right),\;\;\;G=\;\left(\begin{array}{c} \;0.8\\ \;0.6 \end{array}\;\right).$$

The multiplicative noise, ${\left\{\varepsilon_{k}\right\}}_{k\geq 1}$, and the additive noise, ${\left\{\alpha_{k}\right\}}_{k\geq 1}$, are standard white gaussian scalar noises. The initial signal $x_{0}$ is a gaussian two-dimensional random vector with zero mean and covariance matrix $E[x_{0}x_{0}^{T}]=I$. The noise sequences and initial signal vector are assumed to be mutually independent; then, it is easy to see that the signal covariance function is given by $E\left[x_{k}x_{h}^{T}\right]=F_{1}^{k-h}E\left[x_{h}x_{h}^{T}\right],\;h\leq k,$ so assumption ***(A1)*** is satisfied taking $A_{k}=F_{1}^{k}$ and $B_{h}^{T}=F_{1}^{-h}\Sigma_{h}^{x},$ where $\Sigma_{h}^{x}\equiv E\left[x_{h}x_{h}^{T}\right],\;h\geq 1,$ is recursively obtained by:

$$\Sigma_{h}^{x}=F_{1}\Sigma_{h-1}^{x}F_{1}^{T}+F_{2}\Sigma_{h-1}^{x}F_{2}^{T}+GG^{T}\;,\;\;\;h\geq 1;\;\;\;\;\Sigma_{0}^{x}=I.$$

This example shows that assumption ***(A1)*** on the signal autocovariance function is fulfilled by uncertain systems with state-dependent multiplicative noise.

Suppose that a sensor network, comprising 12 sensors, is deployed to measure the stochastic signal $x_{k}$. These sensors are grouped into three clusters and the number of sensors in each cluster is $m_{1}=3$, $m_{2}=4$ and $m_{3}=5$, respectively. Scalar measurements are provided by the 12 sensors, according to model (1), with the following time-invariant observation matrices:

$$C_{k}^{\left(r(1)\right)}=\left[0.8\;\;0.9\right],\;\;\;\;C_{k}^{\left(r(2)\right)}=\left[0.6\;\;0.7\right],\;\;\;\;C_{k}^{\left(r(3)\right)}=\left[0.7\;\;0.8\right],\;\;\;\;r=1,2,3;$$

$$C_{k}^{\left(2(4)\right)}=C_{k}^{\left(3(4)\right)}=\left[0.9\;\;0.5\right],\;\;\;\;\;C_{k}^{\left(3(5)\right)}=\left[0.5\;\;0.5\right].$$

The additive measurement noises are defined as $v_{k}^{\left(r(i)\right)}=v^{\left(r\right)}\eta_{k}^{\left(r\right)}$, for all $i=1,\ldots ,m_{r}$, where $v^{\left(1\right)}=0.4$, $v^{\left(2\right)}=0.7$, $v^{\left(3\right)}=1$, and ${\left\{\eta_{k}^{\left(r\right)}\right\}}_{k\geq 1}$, r=1,2,3, are independent zero-mean Gaussian white process with variance 10; so the measurement noises in each cluster r=1,2,3, are correlated with $R_{k}^{r(ij)}=10{\left(v^{\left(r\right)}\right)}^{2},\;k\geq 1;\;i,j=1,\ldots ,m_{r}.$

Assume that the transmissions to the local processors are subject to linear deception attacks and the data injected by the attackers are described by (2), where the attack noises are defined as $w_{k}^{\left(r(i)\right)}=w^{\left(r\right)}\zeta_{k}^{\left(r\right)}$, for all $i=1,\ldots ,m_{r}$, with $w^{\left(1\right)}=0.1$, $w^{\left(2\right)}=0.25$, $w^{\left(3\right)}=1$, and ${\left\{\zeta_{k}^{\left(r\right)}\right\}}_{k\geq 1}$, r=1,2,3, independent standard Gaussian white processes. Clearly, in each cluster r=1,2,3, the attack noises are correlated with $S_{k}^{r(ij)}={\left(w^{\left(r\right)}\right)}^{2},\;k\geq 1;\;i,j=1,\ldots ,m_{r}.$

According to the theoretical study, for r=1,2,3, the measurements received at the *r*-th local processor are modelled by (3); that is, the attacks are considered to take place randomly and the statuses of the attacks are described by independent sequences of independent Bernoulli random variables with known probabilities $P\left(\lambda_{k}^{\left(r(i)\right)}=1\right)={\bar{\lambda }}^{r(i)},\;k\geq 1,\;r=1,2,3,\;i=1,\ldots ,m_{r}$.

**Figure 1:**
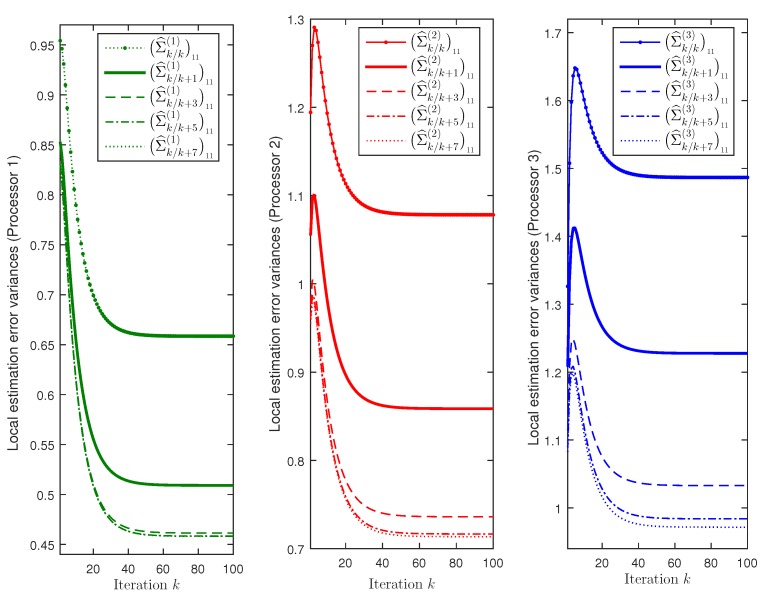
First signal component error variance comparison of the local filters and fixed-point smoothers.

First, considering ${\bar{\lambda }}_{k}^{\left(r(i)\right)}=0.1i$, for r=1,2,3, and $i=1,\ldots ,m_{r}$, the performance of the local filtering estimators has been compared with that of the local fixed-point smoothing estimators; for r=1,2,3, these local estimators are computed in each *r*-th processor using only the measurements received from the sensors of the corresponding *r*-th cluster. For the first signal component, Figure 1 displays the error variances of the local filtering estimators, ${\left({\hat{\Sigma }}_{k/k}^{\left(r\right)}\right)}_{11}$, and the local fixed-point smoothing estimators ${\left({\hat{\Sigma }}_{k/k+N}^{\left(r\right)}\right)}_{11}$, for N=1,3,5,7, are displayed. For the three processors, Figure 1 shows that, as expected, the error variances corresponding to the local smoothers are less than those of the local filters. It is also observed that the accuracy of the smoothers at each fixed-point, *k*, becomes better as the number of available observations, k+N, increases, although this improvement is practically imperceptible for N>3 in processor 1, N>5 in processor 2 and N>7 in processor 3. Note that the best estimation accuracy is obtained in processor 1 and the worst one in processor 3; therefore, we can conclude that, when the filtering performance is better, the improvement of the smoothers is less significant and, actually, it becomes practically negligible for lower values of *N*. Similar results and conclusions are obtained for the second signal component.

**Figure 2:**
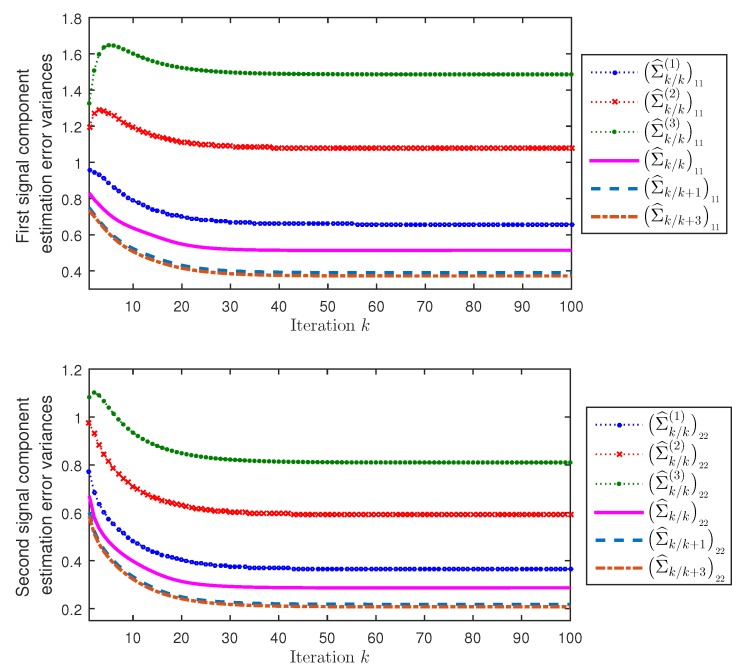
Local filtering and distributed filtering and fixed-point smoothing error variances of the first and second signal components.

Next, considering again ${\bar{\lambda }}_{k}^{\left(r(i)\right)}=0.1i$, for r=1,2,3, and $i=1,\ldots ,m_{r}$, the performance of the distributed filtering and fixed-point smoothing estimators have been compared with that of the local filtering estimators of the three processors. Figure 2 displays, for both first and second signal components (a=1,2), the error variances of the local filtering estimators ${\left({\hat{\Sigma }}_{k/k}^{\left(r\right)}\right)}_{aa}$, r=1,2,3, and the distributed filtering and smoothing estimators ${\left({\hat{\Sigma }}_{k/k+N}\right)}_{aa}$, N=0,1,3. On the one hand, this figure shows that the error variances of the distributed fusion filtering estimators are smaller than those of every local filter; consequently, the distributed fusion filtering estimators outperform all the local ones, agreeing with what theoretically is expected, since all the information of the three clusters is available to the distributed filters, while the local ones are based on the information of a single cluster. On the other hand, agreeing with the comments made about Figure 1, from Figure 2 it is also concluded that the error variances of the distributed fusion smoothers are less than those of the filters and, at each fixed-point, *k*, the performance of the smoothers is better as the number of available observations, k+N, increases (note that, in this case, the improvement is practically imperceptible for N>1).

Finally, assuming the same success probability of attack during the transmissions of the measured outputs of all the sensors in the three clusters, ${\bar{\lambda }}_{k}^{\left(r(i)\right)}=\bar{\lambda }$, r=1,2,3, $i=1,\ldots ,m_{r}$, we analyze how this probability affects the error variances of the distributed filtering estimators. Taking into account that these variances stabilize after a sufficiently large number of iterations, only the results of the last iteration (k=100) are shown in Table 1. Specifically, the distributed filtering error variances of the first and second signal components, ${\left({\hat{\Sigma }}_{100/100}\right)}_{aa}$, a=1,2, together with the corresponding percent variation rates are shown in Table 1, when $\bar{\lambda }$ varies from 0.1 to 0.9. From this table, we conclude that, for both signal components, as $\bar{\lambda }$ increases, the distributed filtering error variances become higher, meaning that, as expected, the lower the probability of successful attacks is, the better estimators are obtained. In addition, for both signal components, it is observed that the deterioration of the estimators is more significant (the percent variation rate of the error variance is larger) for high attack probabilities ($\bar{\lambda }\geq 0.7$).

## 6. Concluding Remark

This paper contributes to the literature on the distributed fusion filtering and fixed-point smoothing problems in clustering sensor networks subject to random deception attacks. Actually, the sensor nodes of the network are grouped into some clusters and each cluster is connected to a local processor, which gathers the measured outputs of all the sensors in the cluster. During this transmission, the measured outputs sent by the sensors to the local processor may be deceptively modified by malicious attackers, who launch random deception attacks with known probabilities of success that may be different at each sensor. Aggregating the information received at every local processor, and using an innovation approach, recursive and easily implementable local filtering and fixed-point smoothing algorithms have been designed without requiring full knowledge of the signal evolution model, but only the first and second order moments of the processes involved in the measurement equations. Once the local estimators are available, they are transmitted to the global fusion center where they are fused to obtain the optimal matrix-weighted fusion filter and fixed-point smoother, under the minimum mean-squared error criterion. The simulation results have illustrated the effectiveness of the proposed algorithms, showing that the distributed fusion estimators outperform the local ones. In addition, the influence of the probability of successful attacks on the estimation performance has been analyzed, concluding that the estimation error variance is larger (and, consequently, worse estimations are obtained) for higher attack probabilities of success. Some new research directions stemming from this work would be:

- Considering other different attack strategies (not necessarily linear ones) to cover more general scenarios in practice. Another interesting generalization would be studying the case where the Bernoulli random variables modelling the success or failure of the attacks are not necessarily independent, but they are correlated at consecutive sampling times or they obey a Markovian dependence structure, for example.
- Addressing the estimation problem in clustering sensor networks with a topology represented by a directed or undirected graph, thus generalizing the current study by allowing the exchange of information between sensors and between clusters.
- Investigating other interesting issues related with clustering sensor networks (cluster consensus, sensor mobility, overlap of clusters, cluster position awareness, efficient and energy, uniform clustering or stability of the cluster, among others).

## Figures and Tables

**Table 1:** Distributed filtering error variances at k=100 and percent variation rates of the first and second signal components under different attack success probabilities $\bar{\lambda }$.

Attack Success Probability $\bar{\lambda }$	0.1	0.2	0.3	0.4	0.5	0.6	0.7	0.8	0.9
**Error variance ${\left({\hat{\Sigma }}_{100/100}\right)}_{11}$**	0.4743	0.5597	0.6428	0.7343	0.8427	0.9810	1.1758	1.4950	2.1877
**Percent variation rate**		18.01	14.85	14.23	14.76	16.41	19.86	27.10	46.38
**Error variance ${\left({\hat{\Sigma }}_{100/100}\right)}_{22}$**	0.2650	0.3122	0.3579	0.4082	0.4675	0.5427	0.6478	0.8180	1.1787
**Percent variation rate**		17.81	14.64	14.05	14.53	16.09	19.37	26.23	44.15
